# A scalable system for the fast production of RNA with homogeneous terminal ends

**DOI:** 10.1080/15476286.2022.2123640

**Published:** 2022-09-19

**Authors:** Yuchen Chen, Yan Cheng, Jinzhong Lin

**Affiliations:** State Key Laboratory of Genetic Engineering, School of Life Sciences, Zhongshan Hospital, Fudan University, Shanghai, China

**Keywords:** In vitro transcription, ribozyme, K-turn, HHV-Kt, Twister-Kt

## Abstract

In vitro transcription (IVT) using T7 RNA polymerase has become the most common method to synthesize RNAs for use in basic research and pharmaceutical applications. To solve the heterogeneity issue associated with the system, cis-acting ribozymes have been exploited to direct co-transcriptional processing to yield target RNAs with precisely defined ends. However, traditionally used ribozymes have many limitations, such as low efficiency and special sequence requirements of target RNAs. In addition, the introduction of ribozymes in IVT complicates the downstream purification of target RNAs. Here we describe a new cassette of engineered ribozymes (HHV-Kt and Twister-Kt) that can work in concert to produce RNA with defined 5’ and 3’ ends. The pair of ribozymes displayed reliably high activity when working with RNA of various lengths and structures. The engineered ribozymes also carry a K-turn RNA motif that enables fast post-IVT clearance of cleaved ribozymes and uncleaved precursors using K-turn affinity resins. Finally, we demonstrated the scalability of our system for the rapid production of large quantities of homogeneous RNA samples.

## Introduction

RNA has attracted increasing research interest due to its variety of biological functions and the great potential for pharmaceutical use. Consequently, RNA production has become a routine and, in some applications, a critical task in many labs or industries. Most techniques are still in development and evolving. There are two ways to synthesize RNA molecules, chemically and enzymatically. Chemical synthesis is preferred for short RNA oligomers with less than 80 nucleotides, but efficiency drops considerably with increased length [1]. Enzymatic synthesis using bacteriophage RNA polymerase such as T7 RNAP has proved powerful for preparing RNA in the laboratory and industrial settings. However, in vitro transcription (IVT) by T7 RNAP has inherent drawbacks. RNA products usually have heterogeneous 5’ and 3’ terminal ends due to non-templated or self-templated additions of nucleotides [2–4]. This is particularly common with the 3’ terminus, which may interfere with downstream studies and applications. In addition, T7 RNAP has specific sequence requirements in the 5’ end of the RNA. To solve this problem, self-cleaving ribozymes were utilized in IVT [5]. When fused with the target RNA at the 5’ or the 3’ end, ribozymes can catalyse the site-specific hydrolysis of a phosphodiester bond, creating defined terminal ends co-transcriptionally. For this purpose, the hammerhead (HH) and the hepatitis delta virus (HDV) ribozymes are the most widely used ones to generate homogeneous RNA in IVT [6]. HH can be put upstream or downstream of target RNAs. It does not have sequence requirements for the RNA when used at the 5’ terminus. However, when placed at the 3’ terminus of target RNAs, the target RNA must end with GUC triplets for the active cleavage to occur [7]. HDV ribozyme does not have this restriction and can be used as a 3’ ribozyme [8,9].

Although effective in generating homogeneous RNA, introducing ribozymes creates a situation where downstream purification of the target RNA becomes challenging. Two major side-products must be removed, the released ribozyme and the uncleaved precursor. In practice, IVT reactions are usually subject to denaturing gel electrophoresis to separate the target RNA from unwanted species based on their sizes [10]. This time-consuming method only works with RNA of fewer than 500 nucleotides, which also should considerably differ in size from the ribozyme to achieve reasonable separation in the denaturing gel. After purification by denaturing gel, there is also the risk that RNAs will misfold rather than refold to the native state.

HH and HDV ribozymes were first introduced in the application of homogeneous RNA production in the nineties of the last century when a very limited number of ribozymes were discovered [6,11]. This cassette has been used for several decades, and very few improvements have been made to the system. These two ribozymes do not consistently deliver high cleavage efficiency in real-world applications. Especially when the target RNA has a complex secondary and tertiary structure, it may interfere with the correct folding of the ribozyme, rendering it inactive.

Recently, more ribozymes have been discovered and characterized [12,13]. We decided to expand the repertoire of ribozymes for use in IVT, aiming to develop a highly efficient system for manufacturing high-quality RNA to meet both laboratory and industrial needs. Among the ribozymes screened, we found that a hammerhead variant (HHV) and a twister ribozyme performed best in producing defined 5’ and 3’ terminal ends. On top of that, we introduced a K-turn RNA motif to each ribozyme to create the HHV-Kt and Twister-Kt, respectively. We demonstrated that this new cassette of engineered ribozymes delivered consistently high cleavage efficiency for a broad kind of RNA molecules, from short small RNA species to long RNA fragments of complex structures. Furthermore, the introduction of K-turn enables fast elimination of the ribozyme and other side products from IVT reactions. RNA of high purity and homogeneity can be obtained by one-step post-IVT purification, which can be scaled up on a chromatography system.

## Results

### Characterization of new 5’ and 3’ ribozymes

**Figure 1.**
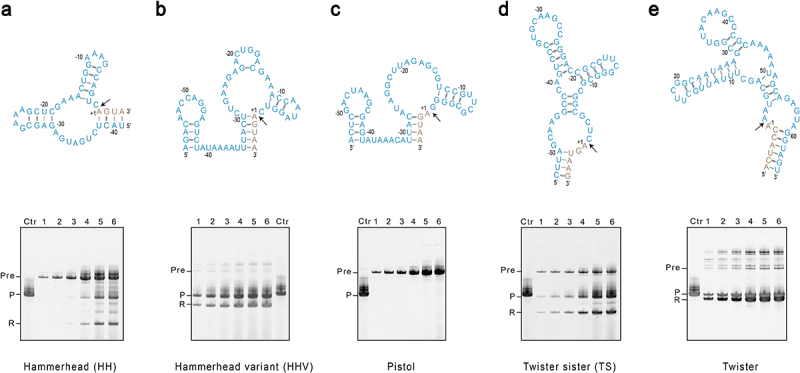
In-vitro synthesis of mt-tRNA^Met^ assisted by cis-acting ribozymes. A ribozyme was placed either upstream of mt-tRNA^Met^ as for (A) hammerhead (HH), (B) Hammerhead variant (HHV), (C) Pistol, and (D) Twister sister (TS), or downstream of mt-tRNA^Met^ as for (E) twister. Sequences and secondary structures for each ribozyme are shown in blue on top of each panel. Sequences from tRNA-tRNA^Met^ are shown in grey, and only the terminal ends are displayed. The black arrow indicates the cleavage site. Three independent IVT reactions were set up for each construct, and samples from different time points were resolved by denaturing gel electrophoresis. A typical experiment was shown at the bottom. Lanes 1–6 are samples taken at 1, 2, 3, 6, 12, and 24 hours of the transcription reaction. Lane Ctr is the control mt-tRNA^Met^ in vitro transcribed without fusing with ribozymes. Pre stands for uncleaved precursor, P strands for the product mt-tRNA^Met^, and R stands for the freed ribozyme.

Four recently discovered ribozymes, Hammerhead Variant (HHV), Twister Sister (TS), Pistol, and Twister, appeared to have high activity [12,13]. We set out to explore their application in homogenous RNA production as alternatives to traditional HH or HDV ribozymes. Using the structured human mitochondrial tRNA^Met^ (mt-tRNA^Met^) as a model system, we characterized the efficiency and sequence requirements of these ribozymes. We fused wild-type ribozymes with the mt-tRNA^Met^ sequence and placed them under the T7 promoter for IVT. HHV, TS, and Pistol were placed upstream of the tRNA for producing a defined 5’ end, while Twister was installed downstream of the tRNA to make a defined 3’ end. A base-paring stem structure formed between the ribozyme and the target RNA is a conserved feature for most ribozymes and is required for maximum efficiency (Fig. 1). For comparison, we also tried originally-used HH to make mt-tRNA^Met^ .

An IVT reaction was set up for each transcript and incubated at 37°C for 24 hours. RNA production was monitored by taking samples at different time points and resolving them by denaturing polyacrylamide gel electrophoresis (Fig. 1). For each construct, three independent IVT experiments were carried out for the statistical analysis of cleavage efficiency based on the gel band intensities (Supplementary Fig. S1a). Transcription reached a plateau after 3 hours for HHV-tRNA and tRNA-Twister. Efficient co-transcriptional cleavage of HHV and Twister occurred from the very beginning of IVT, generating three main RNA species corresponding to full-length precursor, cleaved ribozyme, and the product mt-tRNA^Met^. Nearly all mt-tRNA^Met^ were released from the HHV-tRNA precursor. There is a persistence of minor uncleaved tRNA-Twister precursor throughout the reaction, which did not build up over time. For TS-tRNA, the uncleaved precursor is prominent. In the first three hours, about 60% of TS-tRNA was cleaved. Prolonged incubation yielded more of the total RNA and resulted in a higher level of cleavage. After 24 hours, TS-tRNA precursor accounted for ~22% of the total RNA. We only observed Pistol-tRNA precursor, suggesting Pistol could not fold into an active conformation when fused with mt-tRNA^Met^ (Fig. 1C). As expected, the traditional HH ribozyme did not show high cleavage efficiency. A slow release of HH occurred after 3 hours of transcription, and less than 50% of total HH-tRNA was cleaved when IVT was finished.

**Figure 2.**
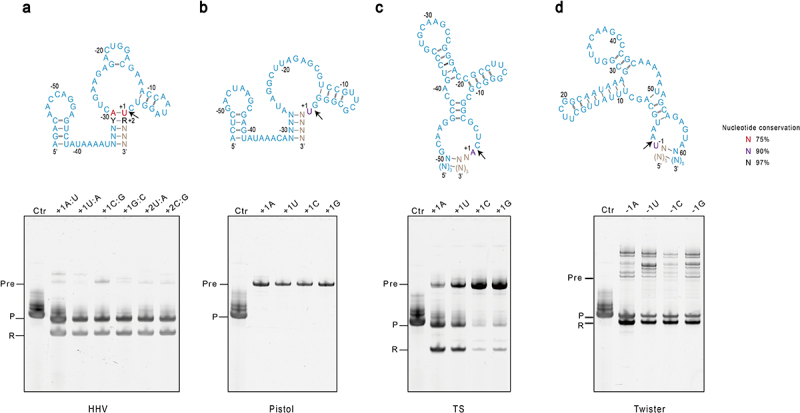
Characterization of sequence requirements at the catalytic sites of ribozymes. The constructs to produce mt-tRNA^met^ are the same as in Fig. 1, except that the conserved nucleotides at the catalytic sites are mutated. Sequences and secondary structures for (A) HHV, (B) Pistol, (C) TS, and (D) Twister are shown in blue on top of each panel. Conserved residues are indicated. Y: pyrimidine, R: purine. The black arrow indicates the cleavage site. For each ribozyme, various point or base-pair mutations were introduced into the conserved sites, and their effects on the ribozyme activity were tested. The IVT reactions of each construct at three hours were analysed by denaturing gel electrophoresis shown on the bottom panel. Lane Ctr is the control mt-tRNA^Met^ in vitro transcribed without fusing with ribozymes. The label Pre stands for uncleaved precursor, P strands for the product mt-tRNA^met^, and R stands for the cleaved ribozyme.

In ribozymes, the nucleotides near the catalytic core are usually conserved [14], which would demand specific sequence requirements in target RNAs, limiting the usability of ribozymes in the application of homogeneous RNA production. We then examined whether the conserved nucleotides are required for the activity of the ribozymes (Fig. 2 & Supplementary Fig. S1b). In HHV, the nucleotide immediately following the cleavage site (+1 position) is 75% conserved U, while the next following nucleotide (+2 position) is 97% purine [13]. In the above experiment, A instead of U was used at the +1 position since mt-tRNA^Met^ begins with A. The result showed that ribozyme activity was not compromised. We further changed the +1 nucleotide to U, G, and C, and the +2 nucleotide was changed from a purine to a pyrimidine C or U. All these constructs displayed equally high cleavage efficiency, suggesting the residue near the catalytic core of HHV can be well-tolerated, making it an excellent choice for a 5’ ribozyme (Fig. 2A). TS is another 5’ ribozyme in which the +1 nucleotide immediately following the cleavage site is 90% conserved A [13]. Replacement with U resulted in the weaker activity of the ribozyme, while changes to G and C led to a significant drop in the cleavage, suggesting the twister ribozyme prefers +1 A for optimal activity (Fig. 2C). The Pistol ribozyme failed to cleave itself off the precursor in the above experiment. We suspected this might be due to the sensitive +1 nucleotide, which is U found in most pistol sequences, and yet an A nucleotide was used instead in the above experiment to make mt-tRNA^Met^. However, substitutions of A with U, G, and C did not restore the activity (Fig. 2B), and the extension of the P3 stem to up to eight base pairs did not improve its activity either (Supplemental Fig. S2). suggesting mt-tRNA^Met^ may have a negative impact on the activities of the Pistol ribozyme.

Twister is the only ribozyme placed at the 3’ end of target RNA. The −1 nucleotide immediately upstream of the cleavage site is U in the original Twister sequence, which is 90% conserved [12]. Substitution of U with A as in the above mt-tRNA^Met^ did not affect the efficiency (Fig. 1E). Further changes to G or C at this position led to equally high cleavage, suggesting that the twister’s activity is insensitive to the identity of −1 nucleotide, which makes Twister a second universal 3’ ribozyme since the introduction of HDV (Fig. 2D).

### Engineering of ribozymes for fast post-IVT cleanup

As mentioned in the introduction, using ribozymes in the IVT makes downstream purification of the target RNA problematic since it is mixed with unwanted side products, mainly the cleaved ribozyme and the uncleaved precursors. In the case of tRNA, whose size is close to a ribozyme, size-based separation methods are not feasible. To solve this problem, we decided to tag the ribozymes so they can be selectively cleared from the IVT reaction.

**Figure 3.**
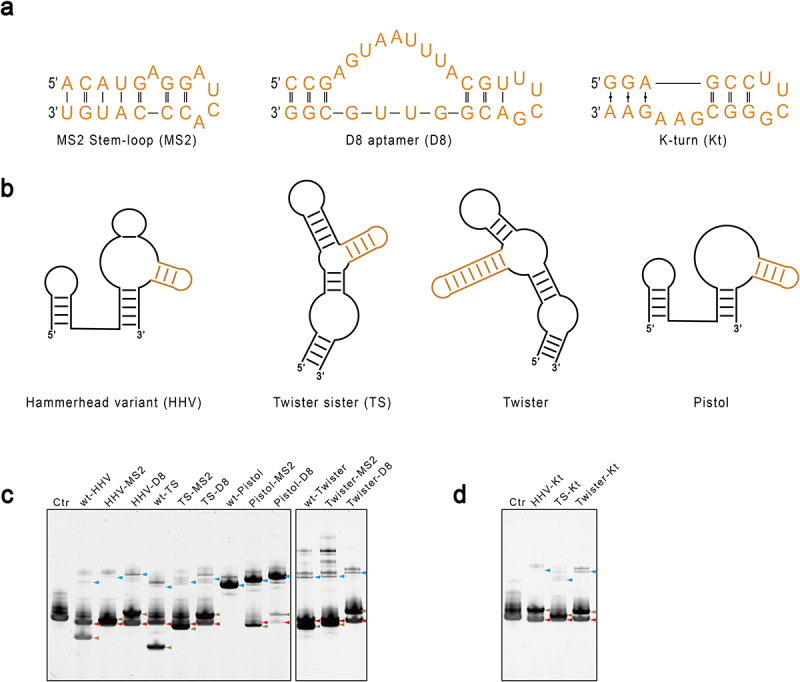
Engineering of ribozymes by introducing an affinity tag to the non-essential stem. A. Sequences of the three RNA affinity tags explored in this study: the MS2 stem-loop (MS2), the D8 aptamer (D8), and the K-turn motif (Kt). B. The affinity tag was attached to the non-essential stem of each ribozyme coloured in Orange. C and D. Activities of the engineered ribozymes. The modified ribozymes were used to produce mt-tRNA^Met^ as in previous experiments, and RNA was analysed by denaturing gel electrophoresis. The red arrow indicates the position of the released mt-tRNA^Met^. The blue arrow points to the precursor, and the brown arrow points to the cleaved ribozymes.

We screened three options of RNA motifs that can be used as affinity tags: an MS2 stem-loop that specifically binds the MS2 coat protein [15], a Sephadex-binding RNA aptamer D8 [16], and an RNA K-turn motif that binds to ribosomal protein L7Ae with high-affinity [17] (Fig. 3A). The motif was attached to the non-essential stem structure of ribozymes, as illustrated in Fig. 3B. We used these engineered ribozymes to produce mt-tRNA^Met^ as did in the previous experiments. Interestingly, all engineered ribozymes displayed better cleavage efficiency than the original ones (Fig. 3C). This is best manifested by the pistol ribozyme, which was inactive in the Pistol-tRNA transcript, but weak cleavage took place for both MS2- and D8- modified ribozymes. This indicates that the non-essential stem may play a structural role in promoting the correct folding of the ribozyme.

**Figure 4.**
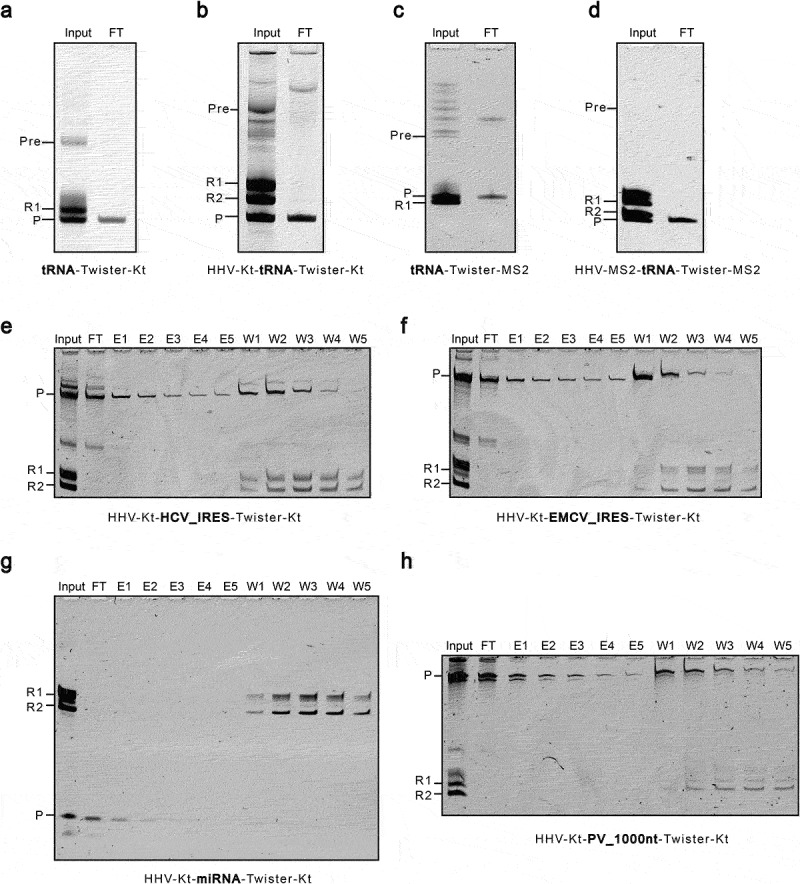
Post-transcriptional clean-up of IVT reactions using K-turn or MS2 affinity resins. A and B. K-turn modified ribozymes, HHV-Kt and Twister-Kt, were used to produce mt-tRNA^Met^ with defined ends. C and D. MS2 modified ribozymes, HHV-MS2 and Twister-MS2, were used to produce mt-tRNA^Met^ with defined ends. e-h. HHV-Kt and Twister-Kt were used in combination to produce the HCV IRES (E), the EMCV IRES (F), the Let-7 miRNA (G), and a 1KB RNA (H) with homogeneous 5’ and 3’ ends. Input is the IVT reaction before purification with the K-turn or MS2 affinity resin. FT is the flow-through of the resin. For HCV IRES, EMCV IRES, miRNA, and 1KB RNA, additional elution with binding buffer was performed (E1-E5). The RNA remaining on the resin was washed off with stripping buffer (W1-W5). Pre is the precursor transcript. R1 and R2 denote the Twister and HHV ribozymes, respectively. P is the released RNA product.

Following transcription, IVT reactions were directly applied onto a spin column loaded with relevant affinity resins or beads, and the flow-through was collected for analysis. For MS2-modified ribozyme, the affinity resin was made by conjugating the recombinant MS2 coat protein (MCP) to the CNBr-activated beads. For K-turn modified ribozyme, we conjugated the recombinant L7Ae protein from *Sulfolobus solfataricus* to the CNBr-activated beads. For the D8 aptamer, the Sephadex resin was directly used. Both the K-turn and MS2 affinity resins absorbed modified ribozymes from the IVT reaction with excellent resolution, leaving essentially pure mt-tRNA^Met^ in the flow-through (Fig. 4 A and C). However, the Sephadex beads did not separate the D8-modified ribozyme from the tRNA (Supplemental Fig. S3). We extended the experiment by using either the HHV-Kt/Twister-Kt pair or the HHV-MS2/Twister-MS2 pair to synthesize mt-tRNA^Met^ with defined 5’ and 3’ ends. The 3’ end of Twister was intentionally extended by 10 nucleotides to distinguish its migration from HHV in the denaturing PAGE. As shown in Fig. 4 B and D, all ribozymes cleaved themselves off the precursors independently with high efficiency. Nevertheless, there remained a mixture of longer transcripts corresponding to the uncleaved or partially cleaved precursors. After cleanup with affinity resin, these contaminants, along with the cleaved ribozymes, were removed. Overall, the MS2 affinity resin yielded slightly less tRNA product than the K-turn resin.

### HHV-kt and Twister-kt are compatible with diverse types of RNA

Based on previous results, the HHV-Kt and Twister-Kt pair has emerged as a promising cassette of ribozymes that can be used in homogeneous RNA production. We next used this ribozyme cassette to synthesize more RNA species of diverse types. These include a 1000-nt RNA sequence taken from the 5’-end of the *Poliovirus* genome (NC_002058), a highly structured 554-nt EMCV IRES from the encephalomyocarditis virus [18], the 341-nt HCV IRES from the hepatitis C virus that is partially folded with a dynamic feature [19], and a 22-nt small microRNA let-7 miRNA [20]. For each RNA, HHV-kt and Twister-kt were used simultaneously to generate defined 5’ and 3’ ends (Fig. 4E-H). Following transcription, reactions were applied onto K-turn affinity resins, and the flow-through was collected. Further washes of the resin with binding buffer increased the yield of target RNA. The resins were finally stripped by basic and high salt buffer (0.2 mM Tris base, 2000 mM KCl), and as expected, mainly ribozymes and related side products were found in the elutes. In summary, ribozymes at both ends function properly and efficiently for all three RNA species. The K-turn affinity resins effectively cleared up the undesired ribozyme-containing products. Following this one-step purification, the target RNAs are virtually homogeneous with regard to both purity and terminal ends.

### Large-scale RNA production with reusable K-turn affinity resins

**Figure 5.**
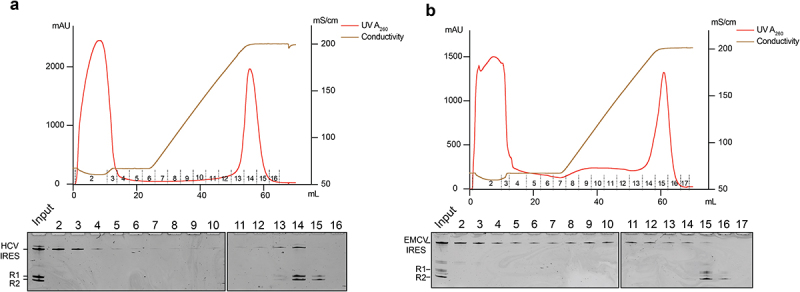
Large-scale production and purification of homogeneous HCV IRES and EMCV IRES with K-turn affinity resins. The ribozyme cassette HHV-Kt and Twister-Kt was used to produce HCV IRES (A) and EMCV IRES(B) with defined 5’ and 3’ ends. Following IVT, RNA was purified on a chromatography system with a prepacked K-turn affinity column. The chromatographic profile was shown on top of each panel. The bottom panel was the denaturing gel electrophoresis analysis of each fraction. R1 and R2 are cleaved Twister-Kt and HHV-Kt, respectively.

Many applications, such as structural analysis and drug development, require large quantities of RNA molecules. Therefore, large-scale RNA production is often necessary. We started to scale up our system to make milligrams of EMCV and HCV IRES RNAs (Fig. 5). A 500 μL of IVT reaction was set up for each RNA, and post-IVT purifications were carried out in a liquid chromatography system equipped with a column prepacked with 1.2 mL of K-turn affinity resin. Factions of flow-through were collected, and the column was then eluted with a gradient of the stripping buffer. A clear separation of the target RNA from unwanted species was achieved for both RNAs in the chromatography system. Over 90% of HCV and EMCV IRES were recovered from IVT, and a total of 0.57 and 0.38 milligrams RNA was obtained for EMCV and HCV IRES, respectively. Theoretically, the system can be further scaled up to produce grams of RNA. Due to the high thermal stability of L7Ae, the K-turn affinity resin was expected to be recycled for repeated use. To demonstrate this, we subjected the resin to consecutive RNA preparations five times and did not observe a decrease in binding capacity (Supplemental Fig. S4).

## Discussion

In vitro transcription by T7 has become the most established method to manufacture RNA, despite synthesized RNA having heterogeneous terminal ends. However, there are many scenarios where RNA of high purity and homogeneity are strictly demanded, for example, in the development of RNA-based therapeutics, such as circular RNA, tRNA, sgRNA, etc. Here we have developed a system that comprises a cassette of engineered ribozymes (HHV-Kt and Twister-Kt) and a novel K-turn affinity resin. HHV-Kt and Twister-Kt working in concert, are capable of generating homogeneous terminal ends for a variety of RNAs, from short linear RNA to large complex RNA molecules. HHV-Kt and Twister-Kt deliver consistently high cleavage efficiency. In addition, HHV-Kt and Twister-Kt do not impose any sequence restrictions on RNA to be synthesized.

In this study, we screened three RNA motifs for an affinity tag and found that the K-turn motif allows effective clearance of ribozymes when IVT is done. The K-turn is a short RNA motif that comprises a three-nucleotide bulge flanked by A•G and G•A basepairs on one side, which can be recognized explicitly by many proteins in nature, including the ribosomal protein L7Ae [17]. Archaeal type L7ae from hyperthermophilic strains *Sulfolobus solfataricus* or *Archaeoglobus fulgidus* can bind K-turn strongly with Kd in the range of picomoles [21]. High affinity and fast kinetics confer L7ae-conjugated resin excellent selectivity against RNA carrying a K-turn motif. Nonetheless, we observed non-specific binding of the target RNA to K-turn affinity resin, which can be alleviated using a stringent buffer condition. In future studies, we will screen L7Ae mutants with reduced non-specific binding, further improving its resolution.

We chose L7ae from a thermophilic archaeon because of its inherited thermal and chemical stability. The resultant K-turn affinity resin can be regenerated with a basic and high-salt solution without losing capacity. Furthermore, we demonstrated that the system could be scaled up to produce milligrams or more of RNA.

Some target RNAs may contain a K-turn motif, so the K-turn purification system is unsuitable. In this case, the HHV-MS2/Twister-MS2 ribozyme cassette can be used with the MS2 affinity resin for RNA production.

Taken together, we have provided a reliable solution for preparing high-quality RNA that can be adapted to different scales.

## Materials and methods

### In-vitro transcription of RNA

All the ribozyme sequences (wildtype and engineered) used in this study were listed in Supplemental Table S1. All DNA sequences used as templates for RNA in-vitro transcription (IVT) were listed in Supplemental Table S2. All DNA was synthesized commercially and cloned into pUC57 vectors under the control of the T7 promoter. DNA templates for transcription were generated by PCR. RNA was prepared by a standard T7 RNAP run-off transcription reaction using the HiScribe™ T7 High Yield RNA Synthesis Kit (New England Biolabs). Typically, a 20 uL reaction was set up according to the instruction and incubated at 37°C for 3 to 24 hours. 2uL of the sample was taken from the reaction at various time points and was analysed by denaturing polyacrylamide gel electrophoresis. For statistical analysis of cleavage efficiency, three independent IVT reactions for each construct were carried out. The band intensities corresponding to precursors, cleaved ribozymes, and released RNA products in denaturing gels were quantified by ImageJ software. The cleavage efficiency was calculated as follows: cleavage efficiency = (cleaved ribozyme + RNA product)/(precursor + cleaved ribozyme + RNA product). The data were plotted as mean ± SD.

### Preparation of K-turn affinity resin

Recombinant L7Ae protein from *Sulfolobus solfataricus* was overexpressed in *E.coli* and purified as previously described [22]. Protein was stored in a buffer containing 10 mM Hepes-KCl, pH 7.5, 160 mM KCl, and 1 mM DTT. To prepare the K-turn affinity resin, L7Aae was conjugated to the CNBr-activated Sepharose™ 4B (Cytiva) according to instructions. Briefly, 20 mg L7Ae protein mixed with 3 mL of the coupling buffer (100 mM NaHCO_3_, pH 8.4, 500 mM NaCl) was incubated with 2.3 mL CNBr-activated Sepharose media. The mixture was then rotated for 2 hours at room temperature for the conjugation to occur. The coupled resins were treated sequentially with the coupling buffer and the blocking buffer (100 mM Tris-Cl, pH 8.0) and washed four times with high pH buffer (100 mM Tris-Cl, pH 8.0, 500 mM NaCl) and low pH buffer ((100 mM NaOAc, pH 4.0, 500 mM NaCl). Finally, the resins were suspended in 50 mM Hepes-K, pH 7.5, and 200 mM KCl, and stored at 4°C.

### RNA clean-up using MS2 affinity resin

Recombinant MS2 coat protein (MCP) carrying a 6xhis tag and MBP fusion protein at its N-terminal was prepared as previously described [23]. To conjugate recombinant MCP to the CNBr-activated Sepharose™ 4B (Cytiva), 30 mg protein was mixed with 4.5 mL of the coupling buffer (100 mM NaHCO_3_, pH 8.4, 500 mM NaCl) and incubated with 3.2 mL CNBr-activated Sepharose medium. The rest of the steps were the same as for preparing the K-turn affinity resin. To purify RNA, 5 µL of IVT reaction was combined with 60 µL resins in a spin column. After centrifugation, the flow-through was collected and saved.

### RNA clean-up using Sephadex G-200

To clear up D8-containing ribozymes, a 15 µL of IVT reaction was brought to 300 µL with the D8 binding buffer (50 mM Hepes-K, pH 7.5, 100 mM NaCl, 10 mM MgCl_2_) and loaded onto 200 µL Sephadex G-200 resin in a spin column. After 20-minute of incubation at room temperature, the resin was precipitated by centrifugation, and the flowthrough was collected. The resin was regenerated by washing twice with 300 µL of 8 M urea.

### RNA clean-up with K-turn affinity resin

To remove RNA carrying the K-turn motif, the IVT reaction was loaded onto the K-turn affinity resins in a spin column pre-equilibrated with binding buffer (25 mM Tris-Cl, pH 7.4, 5 mM MgCl2, 500 mM KCl). After a quick spin, the flowthrough was collected and saved. Target RNA that binds to resin non-specifically can be eluted with additional washes with binding buffer. The K-turn affinity resins were regenerated by washing five times with stripping buffer containing 200 mM Tris base and 2 M KCl and resuspended in binding buffer.

### Large-scale purification of homogeneous RNAs

A prepacked column containing 1.2 mL of K-turn affinity resins was prepared to scale up the RNA production. The column was installed into a liquid chromatography system and equilibrated with the binding buffer (25 mM Tris-Cl, pH 7.4, 500 mM KCl). For RNA purification, a 500 µL of IVT reaction was diluted with 9 mL of binding buffer and applied to the column. Flowthrough was collected and saved. Additional RNA was eluted with a gradient to the stripping buffer (200 mM Tris Base, 2 M KCl). Fractions containing the target RNA were pooled and combined with the flowthrough. The final RNA product was buffer-exchanged to water and concentrated with Amicon® Ultra-15 Centrifugal Filter Devices (Merck Millipore).

## Supplementary Material

Supplemental Material

## Data Availability

The data that support the findings of this study are available from the corresponding author upon request.
