# Supplementary material for: A scalable system for the fast production of RNA with homogeneous terminal ends
Source: RNA Biol. 2022 Sep 19;19(1):1077–84. doi: 10.1080/15476286.2022.2123640 (PMC9521606; doi:10.1080/15476286.2022.2123640)
Supplement: Supplemental Material [file KRNB_A_2123640_SM9505.zip › Revision_Chen_A scalable system for the fast_supplemental_figs.docx]

**Supplemental Figure S1**

**
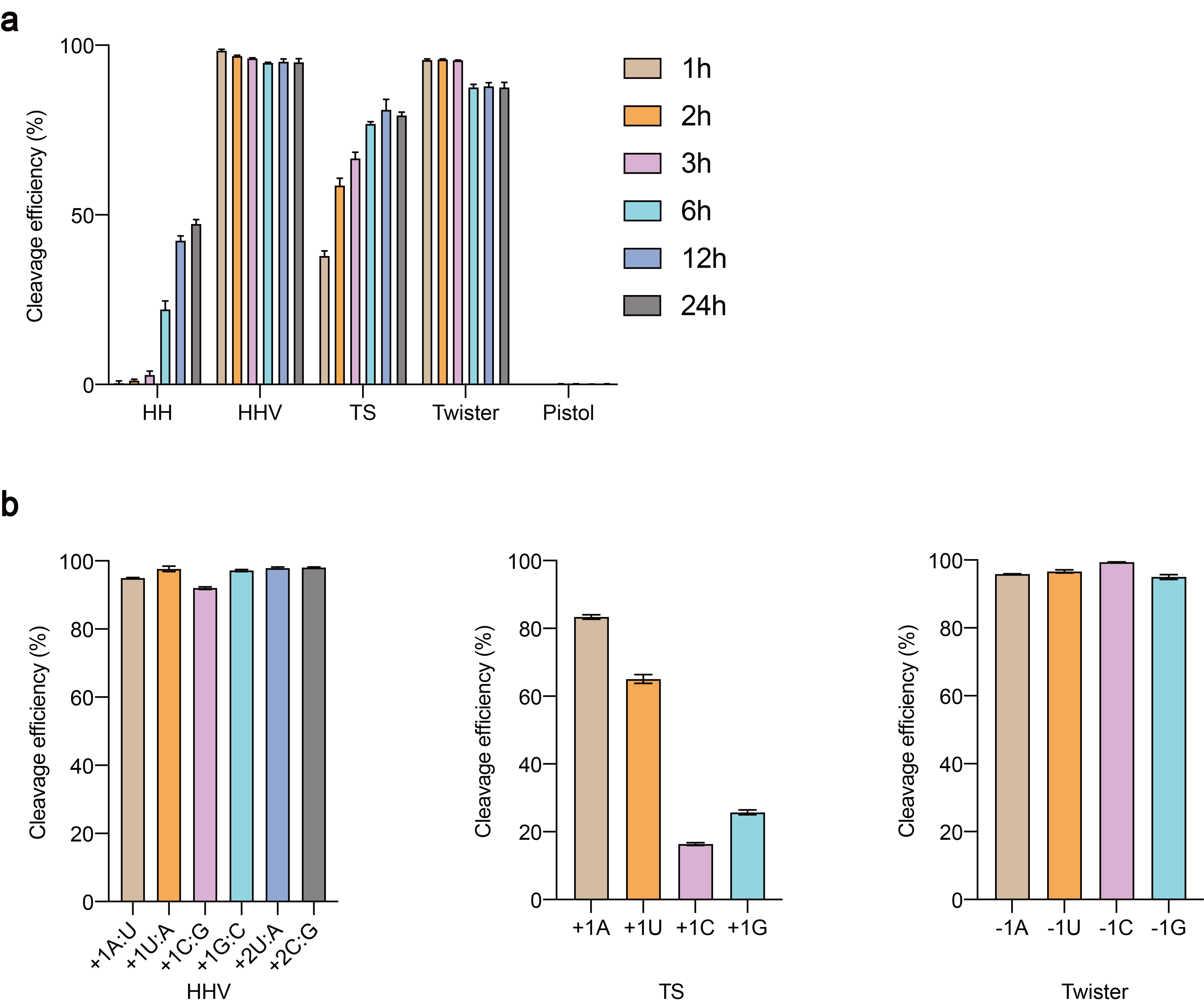
**

**Figure S1: Quantitative analysis of cleavage of ribozymes and ribozyme mutants tested in this study. a.** Cleavage analysis from three independent IVT reactions for each ribozyme corresponding to the experiment in the main text, figure 1. **b.** Cleavage analysis of ribozyme mutants corresponding to the three independent experiments in the main text, figure 2.

**Supplemental Figure S2**

**
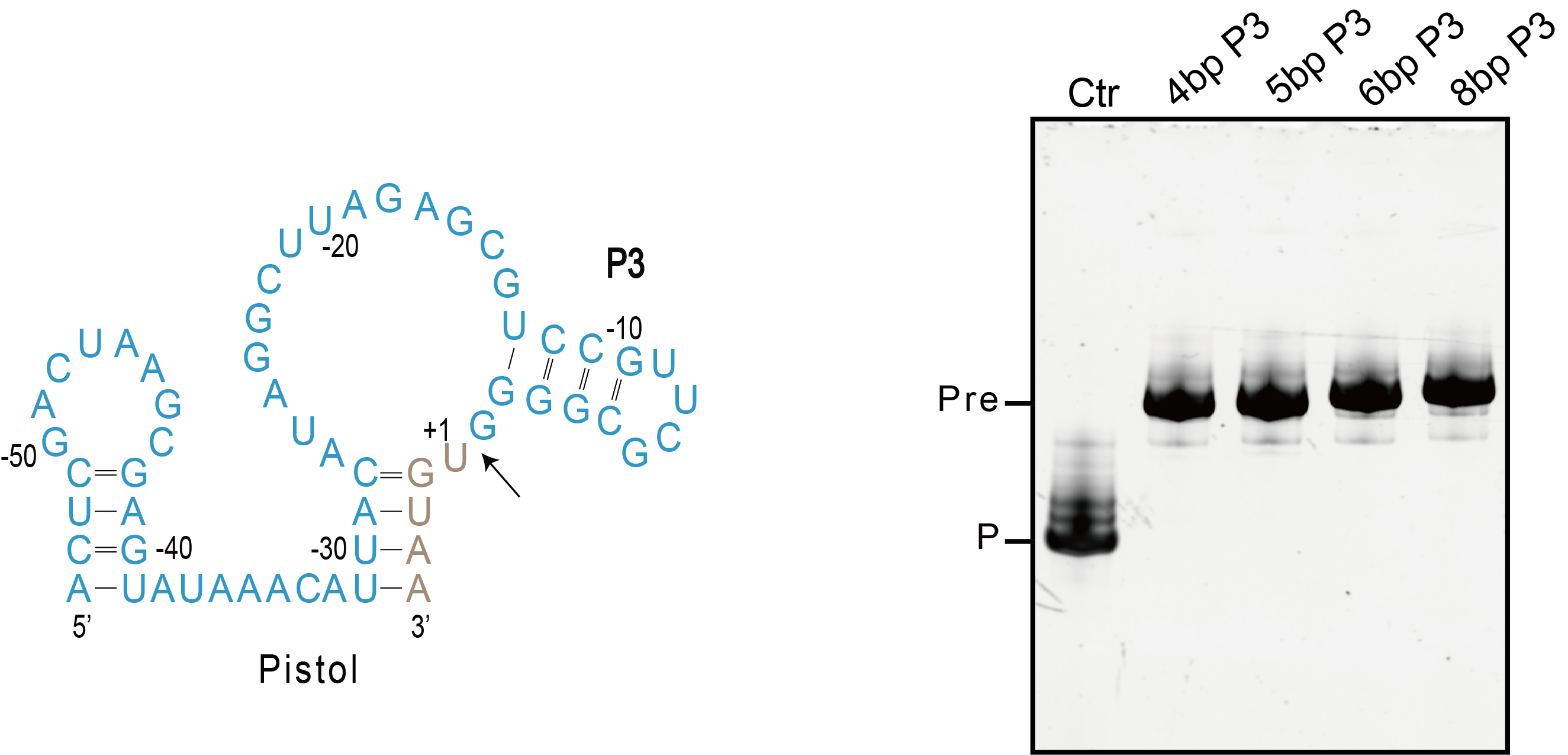
**

**Figure S2: Effect of the P3 stem on the activity of the Pistol ribozyme.** Four constructs of Pistol-tRNA with different lengths of the P3 stem were tested for the activity. After three hours of transcription, the IVT reactions were resolved by denaturing PAGE. Pre stands for the precursor transcript. P denotes the product mt-tRNA^Met^.

**Supplemental Figure S3**


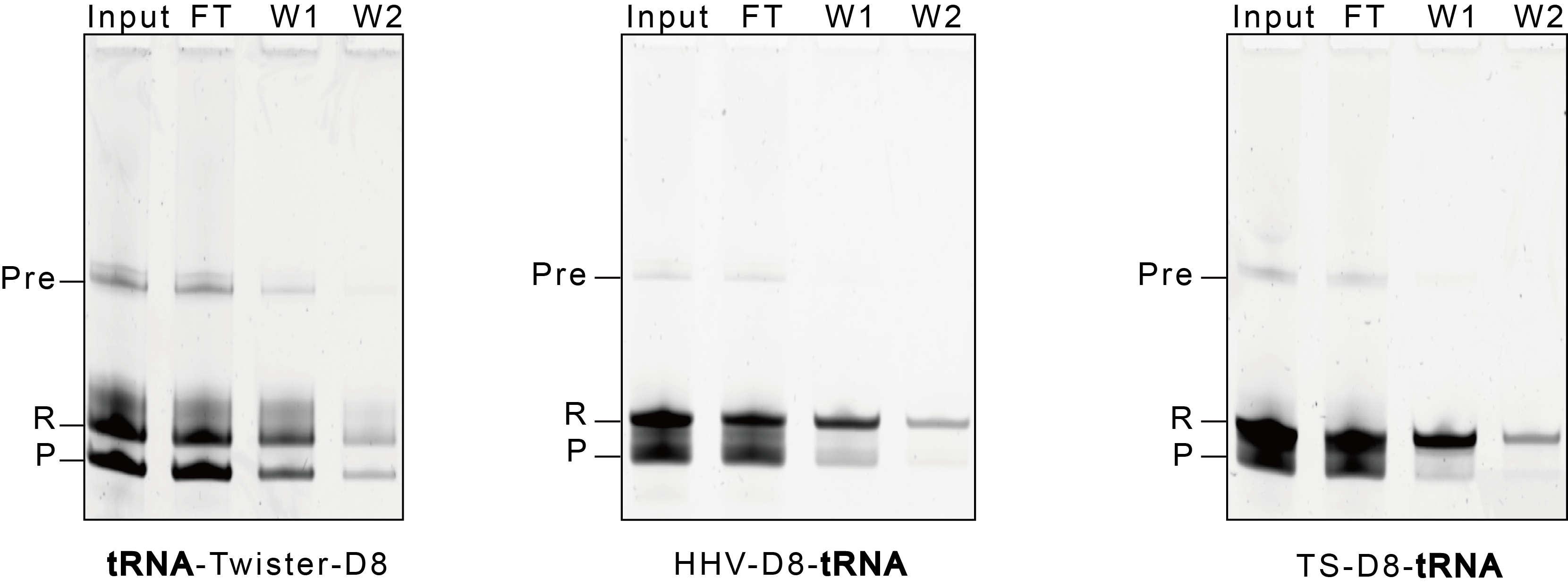


**Figure S3: Post-IVT cleanup of D8 modified ribozymes using the Sephadex resin.**  D8 modified Twister, HHV, and Twister sister were placed at the 5’ or 3’ of mt-tRNA^Met^ for in vitro transcription. The reactions (three hours) were loaded onto Sephadex resins for post-IVT cleanup. Samples from input, flow-through (FT), and washing step (W1, W2) were subjected to denaturing gel electrophoresis. Pre stands for the precursor transcript. R stands for the cleaved ribozyme, and P stands for the product mt-tRNA^Met^.

**Supplemental Figure S4**


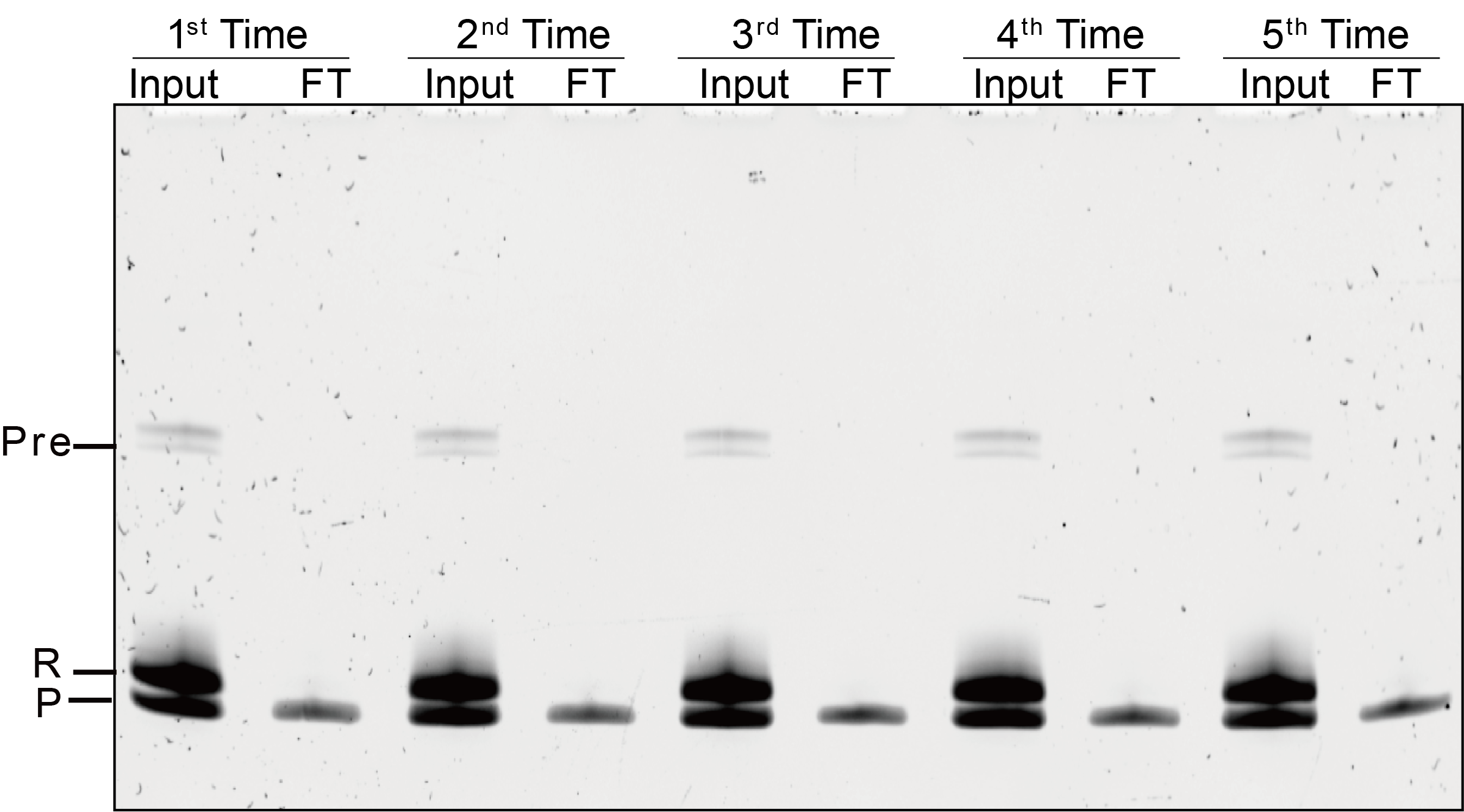


**Figure S4: Characterization of the reusability of the K-turn affinity resin.** The K-turn affinity resin was used to clean up the IVT reactions synthesizing tRNA^Met^-Twister-Kt. After each experiment, the resin was regenerated for the next round of purification. Shown is the result of five consecutive experiments. Pre stands for the precursor transcript. R stands for the cleaved ribozyme, and P stands for the product mt-tRNA^Met^.
