# Supplementary material for: A scalable system for the fast production of RNA with homogeneous terminal ends
Source: RNA Biol. 2022 Sep 19;19(1):1077–84. doi: 10.1080/15476286.2022.2123640 (PMC9521606; doi:10.1080/15476286.2022.2123640)
Supplement: Supplemental Material [file KRNB_A_2123640_SM9505.zip › Revision_Chen_A scalable system for the fast_supplemental_table.docx]

**Supplementary Table 1 Sequences of ribozymes**

| Ribozyme | Sequence |
| --- | --- |
| HH | 5’-NNNNCUGAUGAGAGCGAAAGCUCGAAACUGGAAAGCCAGUCNNNN-3’ |
| HHV | 5’-AGACAACCAGGAGUCUAUAAAAUNNNNNCUGAAGAGACUGGACGAAACCAAUAGGUCNNNNN-3’ |
| HHV-MS2 | 5’-AGACAACCAGGAGUCUAUAAAAUNNNNNCUGAAGAGACUGGACGAAACCACAUGAGGAUCACCCAUGUGGUCNNNNN-3’ |
| HHV-D8 | 5’-AGACAACCAGGAGUCUAUAAAAUNNNNNCUGAAGAGACUGGACGAAACCCGAGUAAUUUACGUUUCGACGGUUGCGGGUCNNNNN-3’ |
| HHV-Kt | 5’-AGACAACCAGGAGUCUAUAAAAUNNNNNCUGAAGAGACUGGACGAAACCAUGGGGAGCCUUCGGGCGAAGAACCAUGGUCNNNNN-3’ |
| TS | 5’-NNNNGCAAGGCCCAGUCCCGUGCAAGCCGGGACCGCCUUCGGGCGCGGCGCUCNNNNNN-3’ |
| TS-MS2 | 5’-NNNNGCAAGGCCCAGUCCCGUGCAAGCCGGGACCGCACAUGAGGAUCACCCAUGUGCGCGGCGCUCNNNNNN-3’ |
| TS-D8 | 5’-NNNNGCAAGGCCCAGUCCCGUGCAAGCCGGGACCGCCCGAGUAAUUUACGUUUCGACGGUUGCGGGCGCGGCGCUCNNNNNN-3’ |
| TS-Kt | 5’-NNNNGCAAGGCCCAGUCCCGUGCAAGCCGGGACCGCUGGGGAGCCUUCGGGCGAAGAACCAGCGCGGCGCUCNNNNNN-3’ |
| Pistol | 5’-ACUCGACUAAGCGAGUAUAAACANNNNAUAGGCUUAGAGCGUCCGUUCGCGGGGNNNNN-3’ |
| Pistol-MS2 | 5’-ACUCGACUAAGCGAGUAUAAACANNNNAUAGGCUUAGAGCGUUGACAUGAGGAUCACCCAUGUCAGGNNNNN-3’ |
| Pistol-D8 | 5’-ACUCGACUAAGCGAGUAUAAACANNNNAUAGGCUUAGAGCGUUGCCGAGUAAUUUACGUUUCGACGGUUGCGGCAGGNNNNN-3’ |
| Twister | 5’-NNNNNNNNNAAUGCAGCUUUAUUGCUUCGGCAAUAAAGCGGUUACAAGCCCGCAAAAAUAGCAGAGUANNNNNNNN-3’ |
| Twister-MS2 | 5’-NNNNNNNNNAAUGCAGCGUACAUGAGGAUCACCCAUGUACGCGGUUACAAGCCCGCAAAAAUAGCAGAGUANNNNNNNN-3 |
| Twister-D8 | 5’-NNNNNNNNNAAUGCAGCUUUCCGAGUAAUUUACGCUUCGGCGGUUGCGGAAAGCGGUUACAAGCCCGCAAAAAUAGCAGAGUANNNNNNNN-3’ |
| Twister-Kt | 5’-NNNNNNNNNAAUGCAGCUUAUGGGGAGCCUUCGGGCGAAGAACCAUAAGCGGUUACAAGCCCGCAAAAAUAGCAGAGUANNNNNNNN-3’ |

N represents A, U, C, or G according to the sequences of ROI. Brown letters are nucleotides in ROI that involve in the formation of active ribozymes.

**Supplementary Table 2 Sequences of the constructs**

| Ribozyme | Sequence |
| --- | --- |
| HH-mt_tRNA^Met^ | 5’-TACTCTGATGAGAGCGAAAGCTCGAAACTGGAAAGCCAGTCAGTAAGGTCAGCTAAATAAGCTATCGGGCCCATACCCCGAAAATGTTGGTTATACCCTTCCCGTACTACCA-3’ |
| HHV-mt_tRNA^Met^ | 5’-AGACAACCAGGAGTCTATAAAATTTACTCTGAAGAGACTGGACGAAACCAATAGGTCAGTAAGGTCAGCTAAATAAGCTATCGGGCCCATACCCCGAAAATGTTGGTTATACCCTTCCCGTACTACCA-3’ |
| HHV_MS2-mt_tRNA^Met^ | 5’-AGACAACCAGGAGTCTATAAAATTTACTCTGAAGAGACTGGACGAAACCACATGAGGATCACCCATGTGGTCAGTAAGGTCAGCTAAATAAGCTATCGGGCCCATACCCCGAAAATGTTGGTTATACCCTTCCCGTACTACCA-3’ |
| HHV_D8-mt_tRNA^Met^ | 5’-AGACAACCAGGAGTCTATAAAATTTACTCTGAAGAGACTGGACGAAACCCGAGTAATTTACGTTTCGACGGTTGCGGGTCAGTAAGGTCAGCTAAATAAGCTATCGGGCCCATACCCCGAAAATGTTGGTTATACCCTTCCCGTACTACCA-3’ |
| HHV_Kt-mt_tRNA^Met^ | 5’-AGACAACCAGGAGTCTATAAAATTTACTCTGAAGAGACTGGACGAAACCATGGGGAGCCTTCGGGCGAAGAACCATGGTCAGTAAGGTCAGCTAAATAAGCTATCGGGCCCATACCCCGAAAATGTTGGTTATACCCTTCCCGTACTACCA-3’ |
| TS-mt_tRNA^Met^ | 5’-CTTAGCAAGGCCCAGTCCCGTGCAAGCCGGGACCGCCTTCGGGCGCGGCGCTCAGTAAGGTCAGCTAAATAAGCTATCGGGCCCATACCCCGAAAATGTTGGTTATACCCTTCCCGTACTACCA-3’ |
| TS_MS2-mt_tRNA^Met^ | 5’-CTTAGCAAGGCCCAGTCCCGTGCAAGCCGGGACCGCACATGAGGATCACCCATGTGCGCGGCGCTCAGTAAGGTCAGCTAAATAAGCTATCGGGCCCATACCCCGAAAATGTTGGTTATACCCTTCCCGTACTACCA-3’ |
| TS_D8-mt_tRNA^Met^ | 5’-CTTAGCAAGGCCCAGTCCCGTGCAAGCCGGGACCGCCCGAGTAATTTACGTTTCGACGGTTGCGGGCGCGGCGCTCAGTAAGGTCAGCTAAATAAGCTATCGGGCCCATACCCCGAAAATGTTGGTTATACCCTTCCCGTACTACCA-3’ |
| TS_Kt-mt_tRNA^Met^ | 5’-CTTAGCAAGGCCCAGTCCCGTGCAAGCCGGGACCGCTGGGGAGCCTTCGGGCGAAGAACCAGCGCGGCGCTCAGTAAGGTCAGCTAAATAAGCTATCGGGCCCATACCCCGAAAATGTTGGTTATACCCTTCCCGTACTACCA-3’ |
| Pistol_mt_tRNA^Met^ | 5’-ACTCGACTAAGCGAGTATAAACATTACATAGGCTTAGAGCGTCCGTTCGCGGGGAGTAAGGTCAGCTAAATAAGCTATCGGGCCCATACCCCGAAAATGTTGGTTATACCCTTCCCGTACTACCA-3’ |
| Pistol_MS2-mt_tRNA^Met^ | 5’-ACTCGACTAAGCGAGTATAAACATTACATAGGCTTAGAGCGTTGACATGAGGATCACCCATGTCAGGAGTAAGGTCAGCTAAATAAGCTATCGGGCCCATACCCCGAAAATGTTGGTTATACCCTTCCCGTACTACCA-3’ |
| Pistol_D8-mt_tRNA^Met^ | 5’-ACTCGACTAAGCGAGTATAAACATTACATAGGCTTAGAGCGTTGCCGAGTAATTTACGTTTCGACGGTTGCGGCAGGAGTAAGGTCAGCTAAATAAGCTATCGGGCCCATACCCCGAAAATGTTGGTTATACCCTTCCCGTACTACCA-3’ |
| Pistol(+1U)-5bp_P3 | 5'-ACTCGACTAAGCGAGTATAAACATTACATAGGCTTAGAGCGTCCGATTCGTCGGGGTGTAAGGTCAGCTAAATAAGCTATCGGGCCCATACCCCGAAAATGTTGGTTATACCCTTCCCGTACTACCA-3' |
| Pistol(+1U)-6bp_P3 | ACTCGACTAAGCGAGTATAAACATTACATAGGCTTAGAGCGTCCGACTTCGGTCGGGGTGTAAGGTCAGCTAAATAAGCTATCGGGCCCATACCCCGAAAATGTTGGTTATACCCTTCCCGTACTACCA |
| Pistol(+1U)-8bp_P3 | ACTCGACTAAGCGAGTATAAACATTACATAGGCTTAGAGCGTCCGACAGTTCGCTGTCGGGGTGTAAGGTCAGCTAAATAAGCTATCGGGCCCATACCCCGAAAATGTTGGTTATACCCTTCCCGTACTACCA |
| Twister_mt_tRNA^Met^ | 5’-AGTAAGGTCAGCTAAATAAGCTATCGGGCCCATACCCCGAAAATGTTGGTTATACCCTTCCCGTACTACCAAATGCAGCTTTATTGCTTCGGCAATAAAGCGGTTACAAGCCCGCAAAAATAGCAGAGTAGGTAGTAC-3’ |
| Twister_MS2-mt_tRNA^Met^ | 5’-AGTAAGGTCAGCTAAATAAGCTATCGGGCCCATACCCCGAAAATGTTGGTTATACCCTTCCCGTACTACCAAATGCAGCGTACATGAGGATCACCCATGTACGCGGTTACAAGCCCGCAAAAATAGCAGAGTAGGTAGTAC-3 |
| Twister_D8-mt_tRNA^Met^ | 5’-AGTAAGGTCAGCTAAATAAGCTATCGGGCCCATACCCCGAAAATGTTGGTTATACCCTTCCCGTACTACCAAATGCAGCTTTCCGAGTAATTTACGCTTCGGCGGTTGCGGAAAGCGGTTACAAGCCCGCAAAAATAGCAGAGTAGGTAGTAC-3’ |
| Twister_Kt-mt_tRNA^Met^ | 5’-AGTAAGGTCAGCTAAATAAGCTATCGGGCCCATACCCCGAAAATGTTGGTTATACCCTTCCCGTACTACCAAATGCAGCTTATGGGGAGCCTTCGGGCGAAGAACCATAAGCGGTTACAAGCCCGCAAAAATAGCAGAGTAGGTAGTAC-3’ |
| HHV_MS2-mt_tRNA^Met^-Twister_MS2 | 5'-AGACAACCAGGAGTCTATAAAATTTACTCTGAAGAGACTGGACGAAACCACATGAGGATCACCCATGTGGTCAGTAAGGTCAGCTAAATAAGCTATCGGGCCCATACCCCGAAAATGTTGGTTATACCCTTCCCGTACTACCAAATGCAGCGTACATGAGGATCACCCATGTACGCGGTTACAAGCCCGCAAAAATAGCAGAGTAGGTAGTAC-3' |
| HHV_Kt-mt_tRNA^Met^-Twister_Kt | 5’-AGACAACCAGGAGTCTATAAAATTTACTCTGAAGAGACTGGACGAAACCATGGGGAGCCTTCGGGCGAAGAACCATGGTCAGTAAGGTCAGCTAAATAAGCTATCGGGCCCATACCCCGAAAATGTTGGTTATACCCTTCCCGTACTACCAAATGCAGCTTATGGGGAGCCTTCGGGCGAAGAACCATAAGCGGTTACAAGCCCGCAAAAATAGCAGAGTAGGTAGTAC-3’ |
| HHV_Kt-miRNA-Twister_Kt | 5’-AGACAACCAGGAGTCTATAAAATCATAGCTGAAGAGACTGGACGAAACCATGGGGAGCCTTCGGGCGAAGAACCATGGTCCTATGCAATTTTCTACCTTACCAATGCAGCTTATGGGGAGCCTTCGGGCGAAGAACCATAAGCGGTTACAAGCCCGCAAAAATAGCAGAGTAGTAAGGTA-3’ |
| HHV_Kt-HCV_IRES-Twister_Kt | 5’-AGACAACCAGGAGTCTATAAAATCTGGCCTGAAGAGACTGGACGAAACCATGGGGAGCCTTCGGGCGAAGAACCATGGTCGCCAGCCCCCTGATGGGGGCGACACTCCACCATGAATCACTCCCCTGTGAGGAACTACTGTCTTCACGCAGAAAGCGTCTAGCCATGGCGTTAGTATGAGTGTCGTGCAGCCTCCAGGACCCCCCCTCCCGGGAGAGCCATAGTGGTCTGCGGAACCGGTGAGTACACCGGAATTGCCAGGACGACCGGGTCCTTTCTTGGATAAACCCGCTCAATGCCTGGAGATTTGGGCGTGCCCCCGCAAGACTGCTAGCCGAGTAGTGTTGGGTCGCGAAAGGCCTTGTGGTACTGCCTGATAGGGTGCTTGCGAGTGCCCCGGGAGGTCTCGTAGACCGTGCACCAATGCAGCTTATGGGGAGCCTTCGGGCGAAGAACCATAAGCGGTTACAAGCCCGCAAAAATAGCAGAGTAGTGCACGG-3’ |
| HHV_Kt-EMCV_IRES-Twister_Kt | 5’-AGACAACCAGGAGTCTATAAAATCGTTACTGAAGAGACTGGACGAAACCATGGGGAGCCTTCGGGCGAAGAACCATGGTCTAACGTTACTGGCCGAAGCCGCTTGGAATAAGGCCGGTGTGCGTTTGTCTATATGTTATTTTCCACCATATTGCCGTCTTTTGGCAATGTGAGGGCCCGGAAACCTGGCCCTGTCTTCTTGACGAGCATTCCTAGGGGTCTTTCCCCTCTCGCCAAAGGAATGCAAGGTCTGTTGAATGTCGTGAAGGAAGCAGTTCCTCTGGAAGCTTCTTGAAGACAAACAACGTCTGTAGCGACCCTTTGCAGGCAGCGGAACCCCCCACCTGGCGACAGGTGCCTCTGCGGCCAAAAGCCACGTGTATAAGATACACCTGCAAAGGCGGCACAACCCCAGTGCCACGTTGTGAGTTGGATAGTTGTGGAAAGAGTCAAATGGCTCTCCTCAAGCGTATTCAACAAGGGGCTGAAGGATGCCCAGAAGGTACCCCATTGTATGGGATCTGATCTGGGGCCTCGGTGCACATGCTTTACATGTGTTTAGTCGAGGTTAAAAAAACGTCTAGGCCCCCCGAACCACGGGGACGTGGTTTTCCTTTGAAAAACACGATGATAATAATGCAGCTTATGGGGAGCCTTCGGGCGAAGAACCATAAGCGGTTACAAGCCCGCAAAAATAGCAGAGTATTATCATC-3’ |
| HHV_Kt-PV_1000nt-Twister_Kt | 5'-AGACAACCAGGAGTCTATAAAATTTTAACTGAAGAGACTGGACGAAACCATGGGGAGCCTTCGGGCGAAGAACCATGGTCTTAAAACAGCTCTGGGGTTGTACCCACCCCAGAGGCCCACGTGGCGGCTAGTACTCCGGTATTGCGGTACCCTTGTACGCCTGTTTTATACTCCCTTCCCGTAACTTAGACGCACAAAACCAAGTTCAATAGAAGGGGGTACAAACCAGTACCACCACGAACAAGCACTTCTGTTTCCCCGGTGATGTCGTATAGACTGCTTGCGTGGTTGAAAGCGACGGATCCGTTATCCGCTTATGTACTTCGAGAAGCCCAGTACCACCTCGGAATCTTCGATGCGTTGCGCTCAGCACTCAACCCCAGAGTGTAGCTTAGGCTGATGAGTCTGGACATCCCTCACCGGTGACGGTGGTCCAGGCTGCGTTGGCGGCCTACCTATGGCTAACGCCATGGGACGCTAGTTGTGAACAAGGTGTGAAGAGCCTATTGAGCTACATAAGAATCCTCCGGCCCCTGAATGCGGCTAATCCCAACCTCGGAGCAGGTGGTCACAAACCAGTGATTGGCCTGTCGTAACGCGCAAGTCCGTGGCGGAACCGACTACTTTGGGTGTCCGTGTTTCCTTTTATTTTATTGTGGCTGCTTATGGTGACAATCACAGATTGTTATCATAAAGCGAATTGGATTGGCCATCCGGTGAAAGTGAGACTCATTATCTATCTGTTTGCTGGATCCGCTCCATTGAGTGTGTTTACTCTAAGTACAATTTCAACAGTTATTTCAATCAGACAATTGTATCATAATGGGTGCTCAGGTTTCATCACAGAAAGTGGGCGCACATGAAAACTCAAATAGAGCGTATGGTGGTTCTACCATTAATTACACCACCATTAATTATTATAGAGATTCAGCTAGTAACGCGGCTTCGAAACAGGACTTCTCTCAAGACCCTTCCAAGTTCACCGAGCCCATCAAGGATGTCCTGATAAAAACAGCCCCAATGCTAAACTCGCCAAACATAGAGGCTTGCGGGTATAGCGATAGAGTACTGCAATTAACAAATGCAGCTTATGGGGAGCCTTCGGGCGAAGAACCATAAGCGGTTACAAGCCCGCAAAAATAGCAGAGTAGTTAATTG-3' |
